# Relationship between eating disorders and internalized problems in chilean adolescents

**DOI:** 10.1186/s40337-021-00474-w

**Published:** 2021-09-26

**Authors:** Alejandra Caqueo-Urízar, Alfonso Urzúa, Jerome Flores, Daniel Acevedo, Jessica Herrera Lorca, Jenifer Casanova

**Affiliations:** 1 Instituto de Alta Investigación, Universidad de Tarapacá, Calle Antofagasta n°1520, Arica, 1000000 Chile; 2 Escuela de Psicología, Universidad Católica del Norte, Antofagasta, Chile; 3 Escuela de Psicología Y Filosofía, Universidad de Tarapacá & Centro Justicia Educacional CJE, Santiago, Chile

**Keywords:** Eating disorder, Internalized problems, Symptomatology, Self-control, Northern Chile

## Abstract

**Background:**

Eating disorders (ED) are associated with internalized problems (INTP), such as depression and anxiety. Evidence shows that ED and INTP are associated with comorbidities. The relationship between these variables has not been the focus of studies on young people from Northern Chile. Children and adolescents are considered as an at-risk group, since they have been found to experience greater vulnerability to psychological problems than other age groups within the population and given the scarcity of studies in Chile, it is necessary to study the relationship between these variables.

**Methods:**

This study analyzed the relationship between ED and INTP in Chilean high school students aged 12 to 18 years using Pearson's correlation. This is a non-experimental and transactional correlational study. We included 2277 students belonging to the public, government-subsidized, and private educational establishments in Northern Chile. The Child and Adolescent Evaluation System [Sistema de Evaluación de Niños y Adolescentes (SENA)] was used to detect a range of INTP, as well as ED. The Brief Self-Control Scale was used in this study.

**Results:**

A strong and direct correlation of the eating disorder variable with depression and anxiety was found. Additionally, significant differences were observed with sex, highlighting females with a higher presence of INTP and ED.

**Conclusions:**

There is a need to establish strategies in the school setting for detecting frequent symptomatology in adolescents with INTP and ED to achieve a timely and accessible intervention.

**Plain English Summary:**

Since the symptomatology of ED is more prevalent in adolescents, this research evaluated relationships between ED and INTP in young people from Northern Chile. This research is relevant because the evidence on this topic in Chile is scarce and the relationships found could be the first research on the subject and serve to design an intervention plan at the school level in the medium and long terms. It is a non-experimental and transactional correlational study because all variables were measured at a single moment, and it sought to establish relationships between variables without assuming causality. The sample of secondary school students consisted of 2277 students, belonging to the public, subsidized, and private schools. Strong relationships were observed between INTP and ED, the strongest correlations being with the variables depression and anxiety. In addition, female participants presented greater problems in ED and INTP.

## Background

The symptomatology of ED is highly prevalent in adolescence and is considered one of the most important precursors of clinical eating behavior disorders [1]. The high stability of ED symptoms together with the significant association with overweight and with a worse mental health prognosis in adulthood, point to the need for early detection and intervention during childhood and adolescence [2]. Studies have shown that frequent symptoms of ED are accompanied by intensified feelings of insecurity, social anxiety, social-emotional isolation, more negative and less positive schemas about the self, lower social competence, and greater interpersonal deficits, which, in addition, are risk and maintenance factors for these disorders [3, 4].

The symptomatology of ED coexists with other pathologies, such as anxiety and depression [5–7]. According to the findings of the World Mental Health Survey, a significant association was observed when models grouping ED with INTP and their subdomains (fear—distress) are analyzed together [8].

Alternatively, INTP can be considered as composed of two sub-factors that explain a more specific grouping; one is using "fear," which encompasses panic disorder, agoraphobia, specific phobia, social phobia, and obsessive–compulsive disorder; the other is through the "distress" sub-factor, including major depressive episode, dysthymia, generalized anxiety disorder, and posttraumatic stress disorder [8]. Consequently, internalized mental health problems include the symptomatology of depression, anxiety, social anxiety, somatic complaints, posttraumatic stress, and obsessive–compulsive disorder [9, 10]. One of the highest prevalence rates of INTP is found in adolescents, affecting 10% to 20% of the general population [11]. Several studies have reported different associations between ED and INTP, thereby indicating psychiatric comorbidity between these mental disorders [12–16].

The last epidemiological study conducted in Chile found that the prevalence of mental health disorders in the infant-juvenile population (4 to 18 years) was 38.3%, and specifically, in adolescents aged 12 to 18 years, the prevalence of anxiety, affective, and ED was 15.7%, 8.6%, and 0.3%, respectively [17]. In the same way, a more current study conducted by Zapata et al. in South Chile reported that 16.1% of adolescents present a risk of developing ED. A higher risk is observed in females (21.8%) than males when the risk is differentiated by sex, whereas the age displaying the highest risk of developing ED was 16 years in females and 17 years in males with an incidence of 25.9% and 9.3%, respectively [18].

Patients with ED have been observed to have a low perception of self-control and a feeling of being out of control in domains such as eating, stress management, relationships with others who are significant, and the way they feel about themselves [19]. Muraven and Baumeister have defined self-control as an exercise of control over the self by the self. That is, self-control occurs when a person attempts to change the way he or she would otherwise think, feel, or behave [20]. Nigg proposed that self-control is an essential aspect of the voluntary process of self-regulation and is also key to understand any psychological problem [21].

An inverse relationship has been observed between self-control and ED, where females with eating disorders present less personal control, which translates into not achieving optimal control of their actions [19, 22].

Children and adolescents are considered as an at-risk group, since they have been found to experience greater vulnerability to psychological problems than other age groups within the population and given the scarcity of studies in this area in Latin America, specifically in Chile, where socioeconomic conditions have undergone significant changes in recent decades, this study establishes the relationship between ED and INTP in students aged 12–18 years of high school in the city of Arica, Chile. These pilot data could be the initial input for designing intervention strategies at the school level.

## Methods

### Study design

This is a non-experimental and transactional correlational study because all variables are measured at a single time, and it seeks to establish relationships between variables without assuming causality [23]. The research objectives were as follows: 1. to observe the correlation between ED and INTP, and 2. to find differences in INTP, conduct problems, and self-control according to sex.

### Participants

Participants were selected through convenience sampling [24] with an age range between 12 and 18 years. The sample consisted of 2277 secondary education students, belonging to the public, subsidized, and private educational establishments. In relation to the Chilean educational system, it generates a certain difference between educational establishments, always having a central element in the way they are financed. This classification includes private establishments, which do not receive state subsidies and cater exclusively to families of a high socioeconomic level, since they cover everything the students need for studying using a monthly payment. Contrarily, there are subsidized schools that can charge families a mandatory fee (up to a certain maximum), without losing the state subsidy, allowing them wide freedom of price range; they can select the students they wish to admit, which is also called a school with shared financing. Most of these schools serve families with middle socioeconomic levels. Further, there are public schools that are free of charge and, ultimately, are obliged to accept all applicants, regardless of the student's background [25].

### Procedure

This study was approved by the Ethics Committee of the Universidad de Tarapacá. To participate in the study, 35 educational establishments in the city of Arica were invited of which 29 agreed to participate in the research. Informed consent was obtained from each parent after explaining the purpose and scope of the study. Further, each student signed an informed consent form before participation.

The evaluations were conducted in groups within each class. At least two trained interviewers answered questions with the teacher of the same course. The duration was approximately 45 min.

It should be noted that during group application, each student had the necessary space to respond without feeling limited or monitored by their peers in their answers.

### Instruments

*Child and Adolescent Assessment System [Sistema de Evaluación de Niños y Adolescentes (SENA)] *[26]: This multidimensional instrument was originally developed in Spanish by experts in psychological assessment and child and adolescent psychopathology. It is designed to detect a wide spectrum of emotional and behavioral problems such as internalized, externalized, contextual, and specific problems in individuals between 3 and 18 years of age. The instrument answered by the students had five options in each response, ordered on a Likert scale ranging from "never or almost never" to "always or almost always." The total of each dimension varied between one and five. The instrument had reliability of 0.7 for its items and scales.

The scale has four general indices, including the problem index, which allowed examining the presence of difficulties and the main disorders evaluated. This has been divided into scales of internalized, externalized, and other problems. For this research, the internalized problem scale was used, which included the following subscales: depression, anxiety, social anxiety, somatic complaints, posttraumatic stress, and obsession-compulsion. The depression subscale was composed of 14 items with reliability of 0.92; anxiety was composed of 10 items with a reliability of 0.87, social anxiety was composed of eight items with a reliability of 0.81, somatic complaints were composed of eight items with a reliability of 0.81, Somatic Complaints is made up of 9 items and has a reliability of (0.82), posttraumatic stress had 11 items with a reliability of 0.82 and obsession-compulsion was made up of six items with a reliability of 0.69. In addition, the scale of other problems was used, focusing on the research on the specific subscale of eating behavior problems, which had a reliability of 0.86, and was made up of 10 items.

*Brief self-control scale *[27]: It is an instrument of 13 Likert-type response items, where 1 is "I do not like it at all" and 5 is "I like it very much" One example of the items was: "Sometimes I cannot stop doing something, even if I know it is wrong or wrong." The original internal consistency analysis of this scale revealed a reliability of 0.85. It was developed from a full 36-item scale. It has been used in adolescents in other countries and is proposed to be unidimensional. This scale was adapted in Spanish and validated in Argentina with a university population, obtaining a reliability of 0.84 in the "Full scale of self-control" and 0.75 in the "Brief scale of self-control" [28].

### Data analysis

Descriptive analysis and analysis of correlations with the study variables were performed using the the Statistical Package for the Social Sciences (SPSS) version 22. Initially, a descriptive analysis of each subscale (such as age, sex, and ethnicity) was estimated, including the mean values and standard deviations, as well as the minimum and maximum ranges obtained, where the skewness and kurtosis indices were used to calculate the normality and distribution of the sample. In addition, assuming normality for the sample size, the Student's t-test was performed for independent samples, using sex as the grouping variable, and Levene's test for homoscedasticity. Subsequently, the Pearson correlation analysis was performed.

## Results

The total sample included 2277 high school students, of whom 1129 (49.6%) students were males and 1148 (50.4%) were females, with a mean age for both sexes was 14.3 ± 1.7 years. In relation to age, 440 (19%) students were 13-years old, followed by 12-years old with 414 (18.2%) students, and 384 (16.9%) students corresponding to 15-years old. Regarding the ethnicities consulted, a large population of students corresponding to 1277 (56.1%) was identified as Latin American, followed by 585 (25.7%) students of Aymara ethnicity (an indigenous people from the Andes and Altiplano regions of South America who have migrated to the cities). Finally, 213 (9.4%) students with the unidentified ethnic group were present.

Of the participants, 933 (41%) students belonged to public schools, 1258 (55.2%) were from subsidized schools, and 86 (3.8%) were from private schools.

Table 1 shows the descriptive statistics for each variable, considering the mean and standard deviation. Both skewness and kurtosis were found to be within the acceptable range to assume normality; hence, it was appropriate to use parametric statistics [29].

**Table 1:** Descriptive statistics

Variables	Min	Max	M	SD	Asymmetry	Kurtosis
Eating disorder	0	98	55.5	11.0	0.70	0.30
Depression	0	104	57.1	13.8	0.89	0.37
Anxiety	0	87	50.7	11.4	0.54	− 0.14
Social anxiety	0	89	52.5	10.6	0.55	0.24
Somatic complaint	0	98	54.8	12.5	0.68	0.27
Posttraumatic stress	0	100	55.3	12.1	0.73	0.33
Obsessive compulsion	0	103	55.4	11.9	0.66	0.40
Self-control	16	50	35.8	6.0	− 0.18	− 0.27

*M* mean, *SD* standard deviation, *Min* minimum, *Max* maximum

Table 2 shows a direct correlation between eating behavior problems and all the variables derived from the internalized problems. A high correlation was found between eating behavior and depression (r = 0.64), moderate correlations with anxiety (r = 0.60), somatic complaints (r = 0.53), posttraumatic stress (r = 0.57), and obsessive compulsion (r = 0.45). Additionally, a low correlation was identified with the social anxiety variable (r = 0.39).

**Table 2:** The Pearson's correlation coefficients (r) between eating disorders and the dimensions of internalized problems and self-control

Variables	1	2	3	4	5	6	7	8
Conduct disorder eating	–	0.640**	0.607**	0.397**	0.537**	0.578**	0.457**	− 0.333**
Depression		–	0.765**	0.477**	0.702**	0.740**	0.522**	− 0.394**
Anxiety			–	0.559**	0.662**	0.746**	0.652**	− 0.314**
Social anxiety				–	0.415**	0.496**	0.477**	− 0.224**
Somatic complaint					–	0.640**	0.492**	− 0.339**
Posttraumatic stress						–	0.607**	− 0.304**
Obsessive compulsion							–	− 0.199**
Self-control								–

** p < 0.001

High correlations were also observed between the variables that make up the internalized problems, such as depression and anxiety (r = 0.76), and the variable somatic complaint with depression (r = 0.70) and anxiety (r = 0.66). At the same time, the variable posttraumatic stress was found to correlate highly with depression (r = 0.74), anxiety (r = 0.74), and somatization (r = 0.64). Obsessive compulsion was moderately correlated with anxiety (r = 0.52) and highly correlated with social anxiety (r = 0.65). A similar correlation was observed between social anxiety and anxiety (r = 0.55). Finally, the self-control variable had a low inverse correlation with the variables, eating behavior problems (r =  − 0.33), depression (r =  − 0.39), anxiety (r =  − 0.31), somatic complaint (r =  − 0.33), posttraumatic stress (r =  − 0.30), and very low correlations with the social anxiety variables (r =  − 0.22), and obsessive compulsion (r =  − 0.19).

Table 3 shows the comparisons between means obtained from both sexes in relation to internalized problems, eating problems, and self-control. There were significant differences between females and males, especially in the variables of eating problems, depression, anxiety, and social complaints. Only in the self-control variable and the hypothesis of equality in the mean were maintained.

**Table 3:** Student's t-test and differences in means by sex

Variables	Sex	M	SD	ST	T	*p* Value
Conduct disorder eating	Females	58.0**	11.7	0.34	11.57	0.000
	Males	52.8	9.62	0.28		
Depression	Females	59.8**	14.8	0.43	9.49	0.000
	Males	54.4	12.1	0.36		
Anxiety	Females	53.4**	11.8	0.35	11.43	0.000
	Males	48.1	10.3	0.30		
Social anxiety	Females	54.2**	10.8	0.32	7.72	0.000
	Males	50.8	10.0	0.29		
Somatic complaint	Females	57.6**	13.3	0.39	10.90	0.000
	Males	52.0	11.0	0.32		
Posttraumatic stress	Females	57.3**	12.7	0.37	8.11	0.000
	Males	53.2	11.1	0.33		
Obsessive compulsion	Females	56.0**	12.4	0.36	2.68	0.007
	Males	54.7	11.3	0.33		
Self-control	Females	35.9	6.3	0.18	0.40	0.689
	Males	35.8	5.6	0.16		

*M* mean, *DT* standard distribution, *P* statistical significance, *ET* typical error, **p < 0.001

## Discussion

As in other regions of the world, this study showed close relationships between ED and INTP, especially depression and anxiety [15, 16, 30–35].

Similarly, the results also coincided with that observed by Keski-Rahkonen and Mustelin, where somatic complaints coexisted with ED [36].

Additionally, females in this study showed a higher score for ED than males, which has been widely reported in previous research [1, 37–40]. This might be due to the constant pressures that females experience for their body image, pursuing a thin ideal picture [41, 42]. This coincided with the evidence of Caqueo-Urizar et al., in the population of young people from northern Chile, where females with increasing age perceived greater pressure to be thin [43]. Another explanation for this finding was male adolescents tend to minimize symptoms to a greater extent than females, especially in relation to questions about weight control, perhaps because there are fewer social constructions about weight ideals for males [44]. Similarly, the results of this study revealed the predominance of higher scores in females in all dimensions that made up the INTP, which were consistent with the findings of Salavera et al. [11].

Meanwhile, the strong association found between ED and posttraumatic stress was consistent with the findings of Sommer et al., who found that 41.1% of their representative sample from the US population with posttraumatic stress (over 18 years of age) was classified as having maladaptive eating symptoms [45]. Thus, posttraumatic stress might be an important risk factor.

Alternatively, a moderate relationship between ED and obsessive compulsion was observed, which was contrastable with the international literature that has investigated this association. Previous studies have reported that both disorders do not have a high correlation, but rather a phenomenon associated with the presence of similar symptomatology (cognitive symptoms) in the two disorders, which could explain the level of the relationship presented in this research. Therefore, different authors are in favor of the conceptualization of obsessive–compulsive disorder and ED as clinical entities that should be well differentiated [46].

Likewise, the moderate relationship found between ED and social anxiety is consistent with what was observed in the study by Levinson and Rodebaugh conducted in the US, where it was found that the association between social anxiety and ED symptoms is due to a shared prospective vulnerability between both variables [47]. This indicates that the presence of other coincident factors (social appearance, anxiety, and maladaptive perfectionism) would mediate the relationship and therefore, this would define the variation in the associative results found.

Furthermore, an inverse correlation was found between the variable self-control and ED, indicating that people with greater self-control can consider themselves capable of regulating their actions and reduce the risky behaviors associated with eating disorders. Self-Control could become a protective factor, which is in agreement with the findings of studies by Lugli and Vivas in young people in the city of Caracas, Venezuela [19] and González et al. in the city of Maracay, Venezuela [22].

Moreover, studies have found that self-control correlates negatively with emotional problems because good self-control can generate better or optimal regulation of emotions and mood. Other studies have found a relationship between low self-control and anxiety-depressive disorders [48, 49].

The strengths of this study were found in the sampling since it was possible to collect information from a large cohort of children and young adults. Second, since the correlations between INTP and ED in the Northern part of Chile have been less studied, this study has provided a foundation for future research.

This study has some limitations. First, this study had a cross-sectional design that did not allow establishing causal relationships; second, only the self-report of the students were available, without having relevant information from teachers and parents; third, rural schools were not considered, which could add interesting information in future studies. Finally, this study excluded mediating variables that could affect the associations found.

## Conclusions

The results in adolescents between 12 and 18 years of age in this sample showed a positive and significant relationship between ED and all the dimensions that compose the INTP. Furthermore, the findings suggested that this relationship was especially strong in the dimensions of depression and anxiety. Likewise, differences were observed in relation to the sex variable, where females presented higher scores in the variables studied. Future research could clarify the causality of these relationships and the factors that influence gender differences.

Finally, this study proved the need to generate strategies for detecting INTP and ED in adolescents, to achieve early intervention at the school level.

## Data Availability

The datasets generated during and/or analyzed during the current study are available from the corresponding author upon reasonable request.

## Abbreviations

SENA: Child and Adolescent Assessment System (Sistema de Evaluación de Niños y Adolescentes) (Spanish acronym)
SPSS: Statistical Package for Social Sciences
